# Ontogeny of plasma cytokine and chemokine concentrations across the first week of human life

**DOI:** 10.1016/j.cyto.2021.155704

**Published:** 2021-09-28

**Authors:** Kinga K. Smolen, Alec L. Plotkin, Casey P. Shannon, Olubukola T. Idoko, Jensen Pak, Alansana Darboe, Simon van Haren, Nelly Amenyogbe, Scott J. Tebbutt, Tobias R. Kollmann, Beate Kampmann, Al Ozonoff, Ofer Levy, Oludare A. Odumade

**Affiliations:** a Precision Vaccines Program, Division of Infectious Diseases, Boston Children’s Hospital, Boston, MA, USA; b Harvard Medical School, Boston, MA, USA; c PROOF Centre of Excellence, 10th Floor, 1190 Hornby Street, Vancouver, BC V6Z 2K5, Canada; d Vaccines & Immunity Theme, Medical Research Council Unit The Gambia at the London School of Hygiene and Tropical Medicine, Banjul, Gambia; e The Vaccine Centre, Faculty of Infectious and Tropical Diseases, London School of Hygiene and Tropical Medicine, London UK; f Telethon Kids Institute, University of Western Australia, Perth, Western Australia, Australia; g UBC Centre for Heart and Lung Innovation, Vancouver, V6T1Z4 BC, Canada; h Department of Medicine, Division of Respiratory Medicine, UBC, Vancouver, V6T1Z4 BC, Canada; i Broad Institute of MIT & Harvard, Cambridge, USA; j Division of Medicine Critical Care, Boston Children’s Hospital, Boston, MA, USA

**Keywords:** Cytokines, Chemokines, Ontogeny, Infant immune, Innate immune

## Abstract

**Introduction/background & aims:**

Early life is marked by distinct and rapidly evolving immunity and increased susceptibility to infection. The vulnerability of the newborn reflects development of a complex immune system in the face of rapidly changing demands during the transition to extra-uterine life. Cytokines and chemokines contribute to this dynamic immune signaling network and can be altered by many factors, such as infection. Newborns undergo dynamic changes important to health and disease, yet there is limited information regarding human neonatal plasma cytokine and chemokine concentrations over the first week of life. The few available studies are limited by small sample size, cross-sectional study design, or focus on perturbed host states like severe infection or prematurity. To characterize immune ontogeny among healthy full-term newborns, we assessed plasma cytokine and chemokine concentrations across the first week of life in a robust longitudinal cohort of healthy, full-term African newborns.

**Methods:**

We analyzed a subgroup of a cohort of healthy newborns at the Medical Research Council Unit in The Gambia (West Africa; N = 608). Peripheral blood plasma was collected from all study participants at birth (day of life (DOL) 0) and at one follow-up time point at DOL 1, 3, or 7. Plasma cytokine and chemokine concentrations were measured by bead-based cytokine multiplex assay. Unsupervised clustering was used to identify patterns in plasma cytokine and chemokine ontogeny during early life.

**Results:**

We observed an increase across the first week of life in plasma Th1 cytokines such as IFNγ and CXCL10 and a decrease in Th2 and anti-inflammatory cytokines such as IL-6 and IL-10, and chemokines such as CXCL8. In contrast, other cytokines and chemokines (e.g. IL-4 and CCL5, respectively) remained unchanged during the first week of life. This robust ontogenetic pattern did not appear to be affected by gestational age or sex.

**Conclusions:**

Ontogeny is a strong driver of newborn plasma-based levels of cytokines and chemokines throughout the first week of life with a rising IFNγ axis suggesting post-natal upregulation of host defense pathways. Our study will prove useful to the design and interpretation of future studies aimed at understanding the neonatal immune system during health and disease.

## 1. Introduction

The first weeks of life represent a period of great ontogenetic change in immunity as well as marked susceptibility to infection [1,2]. Newborn vulnerability during the first few weeks of life reflects the development of a highly complex immune system [3]. As the neonatal immune system adjusts to its new ex utero environment, it faces many challenges, requiring better understanding and identification of markers associated immune maturation in relation to health and disease.

Cytokines and chemokines are low-molecular-weight proteins and key immune signaling molecules with pleiotropic effects on innate and adaptive immune responses [3–5]. Acting via cognate receptors on leukocytes and other host cells, the network of cytokines and chemokines is referred to as the ‘cytokine milieu’, because its function and effect depend on the target cells in diverse tissues. Cytokines and chemokines regulate complex processes such as inflammation, cellular proliferation, differentiation, and development by targeting specific cells[3].

Human plasma contains basal concentrations of a range of cytokines and chemokines [6,7]. In adults and older children, plasma cytokine and chemokine concentrations are altered by a range of factors such as age [9–14], sex, seasonality, and exercise [15–17], yet our understanding of early life ontogeny of neonatal plasma cytokine and chemokine concentrations is limited. These concentrations can be affected by maturation [16,18 19–23], as well as infectious and/or inflammatory states [24–30].

Interest in defining cytokines and chemokines in early life is driven in part by the distinct vulnerability of the newborn. 44% of under 5-year mortality occurs within the neonatal period, with ~75% of all newborn deaths occurring in the first week of life [31]. Furthermore, the majority of these deaths occur in low- and middle-income countries. Cytokines are key players in the pathology of disease and development of therapeutics[32], therefore, characterizing immune trajectories of healthy full-term infants may provide insight into how the infant immune development shapes the susceptibility to disease and responses to vaccine.

Prior studies of plasma cytokine and chemokine concentrations in infants and/or children have been limited by small sample size (n < 20) [33] as well as confounders such as antibiotic treatment [29,30,34] or premature [35–37] participants. Plasma concentrations of IL-1Ra, CXCL10, and TNF-α demonstrated a 60% decrease in children, aged 0.1–12.8 years of age, over time[16]. Given the rapid changes in human immunity, to our knowledge, no other study has characterized the longitudinal trajectory of plasma cytokines and chemokines over the first week of human life in a large cohort of healthy, full-term infants.

The objective of this study was to characterize the normal ontogeny of plasma cytokines and chemokine concentrations in a healthy, term African infant cohort over the first week of life. Such an effort provides insight into normal development and may inform future studies of variance from usual plasma cytokine/chemokine trajectories in the context of early life disease. Using a standardized, multiplex-based assay, we measured a diverse range of cytokine and chemokine profiles and utilized time series clustering to define ontogenetic trajectories during normal neonatal development. We found that plasma cytokine and chemokine concentrations clustered into distinct kinetic groups, including a rise in IFNγ-related cytokines across the first week, suggesting a coordinated ontogeny across the first week of human life.

## 2. Methods

### 2.1. Study design and sample collection

The clinical protocol for this study EPIC002 has been previously described[38,39]. The study is registered on clinicaltrials.gov as NCT03246230. In brief, mothers-infant pairs were consented and enrolled at time of delivery at the Medical Research Council (MRC) Unit at the London School of Hygiene and Tropical Medicine in The Gambia. HIV- and Hepatitis B-negative mothers 18 years or older, were enrolled. Infants were enrolled if >36 weeks of gestational age (as determined by Ballard scoring), with Apgar scores >8 at 5 min and birth weight >2.5 kg. Peripheral blood samples (max volume 2 ml) were collected from infants using sterile sodium heparin tubes (Becton Dickinson). Four time points were collected: Visit 1 (V1), was collected within the first 24 h of life (Day of Life (DOL)0); while the Visit 2 (V2) sample was collected at either DOL1, or DOL3, or DOL7. Plasma samples were processed for analysis as previously published [40]. All plasma samples were stored at −80 ℃ until use. Local and International IRB committees approved all protocols utilized.

### 2.2. Cytokine and chemokine qualification methods

#### Reagents:

Milliplex Human Cytokine/Chemokine MAGNETIC BEAD Premixed 41 Plex Kit. (Millipore cat. #HCYTMAG-60K-PX41), Dulbecco’s phosphate-buffered saline (dPBS, cat. #14190), and Corning Cell-BIND® 384 well plates (cat. #CLS3764).

#### Cytokine/Chemokine assay:

The Luminex assay was performed across 7 Luminex plates. Plasma was diluted 1:2 in dPBS. Cytokines and chemokines were measured using the 41-plex Millipore Milliplex Map Kit (cat. #HCYTMAG-60K-PX41), which contains; *sCD40L, EGF, FGF-2, Flt-3 ligand, Fractalkine, G-CSF, GM-CSF, GRO (CXCL1), IFN-α2, IFN-γ, IL-1α, IL-1β, IL-1ra, IL-2, IL-3, IL-4, IL-5, IL-6, IL-7, IL-8 (CXCL8), IL-9, IL-10, IL-12 (p40), IL-12 (p70), IL-13, IL-15, IL-17A, IP-10 (CXCL10), MCP-1 (CCL2), MCP-3 (CCL7), MDC (CCL22), MIP-1α (CCL3), MIP-1β (CCL4), PDGF-AB/BB, RANTES (CCL5), TGF-α, TNF-α, TNF-β, VEGF, Eotaxin (CCL11),* and *PDGF-AA*. Plasma samples were assayed using a 384-well plate platform following the manufacturer’s instructions, including the provided controls. Samples were analyzed using a Flexmap 3D system with Luminex xPONENT software (Luminex Corp.; Austin, TX, USA).

The xPONENT software files were processed using the drLumi R package. We fitted 4-parameter logistic, 5-parameter logistic, and exponential functions to the dilution series data for each analyte using the scluminex function implemented in drLumi (drLumi::scluminex()), selecting the best fit function in each case. These were then used to determine the lower and upper limits of detection and quantification for each analyte and plate. Analytes that fell below or above these values were imputed to the lower or upper limit of quantification, respectively. For a given sample and analyte, if readings were <30 beads, concentration values were discarded. Samples with all analytes below the lower limit of detection (LLD) were also excluded from analysis.

### 2.3. Statistical analysis

Cytokine and chemokine values were log transformed. ComBat (R package) was used to carry out batch-correction (normalization) across plates based on plate-specific bias identified by PCA. A universal reference standard sample set was added to each plate and used as a bridge sample set across plates.

Cytokines or chemokines with near-zero variance were identified using the nearZeroVar function from the caret R package (low percentage of unique values; high ratio of the frequency of the first and second most frequent values). Near-zero variance cytokines (IL-2, IL-3, and IL-17A) had few values above their respective LLD and were excluded.

Principal component analysis (PCA) was employed to summarize variability in the log_10_-transformed cytokine concentration and fold change datasets, using R packages FactoMineR_2.3 and factoextra_1.0.7. All cytokines were centered and scaled to unit variance.

Statistical differences in cytokine levels between DOL were assessed using the Wilcoxon signed-rank test. Fold-change values were calculated by indexing untransformed concentration values to DOL0, then log_10_-transforming. P-values < 0.05 were considered significant, and the Holm-Bonferroni method using n = 38 cytokines was used to adjust for multiple comparisons. Associations of cytokines with biological sex, ethnicity, maternal age, and gestational age were tested to assess if there were any other factors with significant effects on cytokine concentrations. Each cytokine was modeled as a dependent variable in separate linear models, with the variable of interest, a Visit term, and an interaction term as independent variables.

To identify groups of cytokine and chemokines with common trajectories across the first week of life, we performed time series clustering. The log_10_-transformed cytokine concentrations were first standardized. Average time series were constructed by taking the mean of each cytokine at each DOL. The distances between cytokine time series were calculated with dynamic time warping, using the R package dtw_1.21–3 [41]. This approach yielded improved time series clusters compared to the Euclidean distance metric. We then performed complete-linkage agglomerative hierarchical clustering on the resulting cytokine distances. Five cluster were identified by maximizing the average silhouette width between clusters.

To assess the robustness of our clustering approach, we repeated the procedure across 1000 bootstrap re-samplings using 90% of the data (random selection of 540 out of the 608 participants), and used the frequencies with which cytokines co-clustered to calculate the final groupings, as previously described[42]. In addition, we compared our clusters to those obtained when carrying out consensus clustering on the two time points from each individual, without averaging, to preserve individual-level information[43]. Both methodologies were found to produced similar cytokine and chemokine clusters.

## 3. Results

### 3.1. Cohort enrollment and baseline characteristics of study participants

608 healthy Gambia mother-newborn pairs from the EPIC-002A cohort were analyzed in this study. The cohort demographic table (Table 1) indicates that the plurality of mothers (29.1%) were 25–29 years old, followed by age 20–24 years old (22.2%) and then age 30–34 (20.7%). An approximately equal ratio of male (50.7%) to female (49.3%) newborns were enrolled. The average birth weight was 3.16 kg. A few (n = 4, 0.7%) preterm newborns (<37 weeks gestation) were enrolled within the cohort, but the majority (86.2%) of newborns were early term (≥37 to <38 weeks gestation) or full term (≥38 to <40 weeks gestation). Breastfeeding was initiated for 88.5% of infants at delivery and continued after the first day of life (>98.8%) until four months of age for infants in this cohort.

### 3.2. Association of cytokine and chemokine concentration and demographic factors

Initially, we investigated the associations between plasma cytokine and chemokine concentrations and physical findings at birth, as these can be early indicators of post-natal conditions (i.e., early onset sepsis). There were no significant differences observed in plasma concentrations cytokines and chemokines in relation to respiratory rate, heart rate, weight, length, head circumference, or temperature at birth (data not shown).

We also examined the correlation between plasma cytokine and chemokine concentrations and demographic clinical data over the first week of life. No significant correlations were observed between demographic parameters such as sex, maternal age, gestational age or breastfeeding with cytokines and chemokines measured at DOL 1, 3, or 7.

### 3.3. Ontogenetic changes across the first week of life

**Fig. 1.**
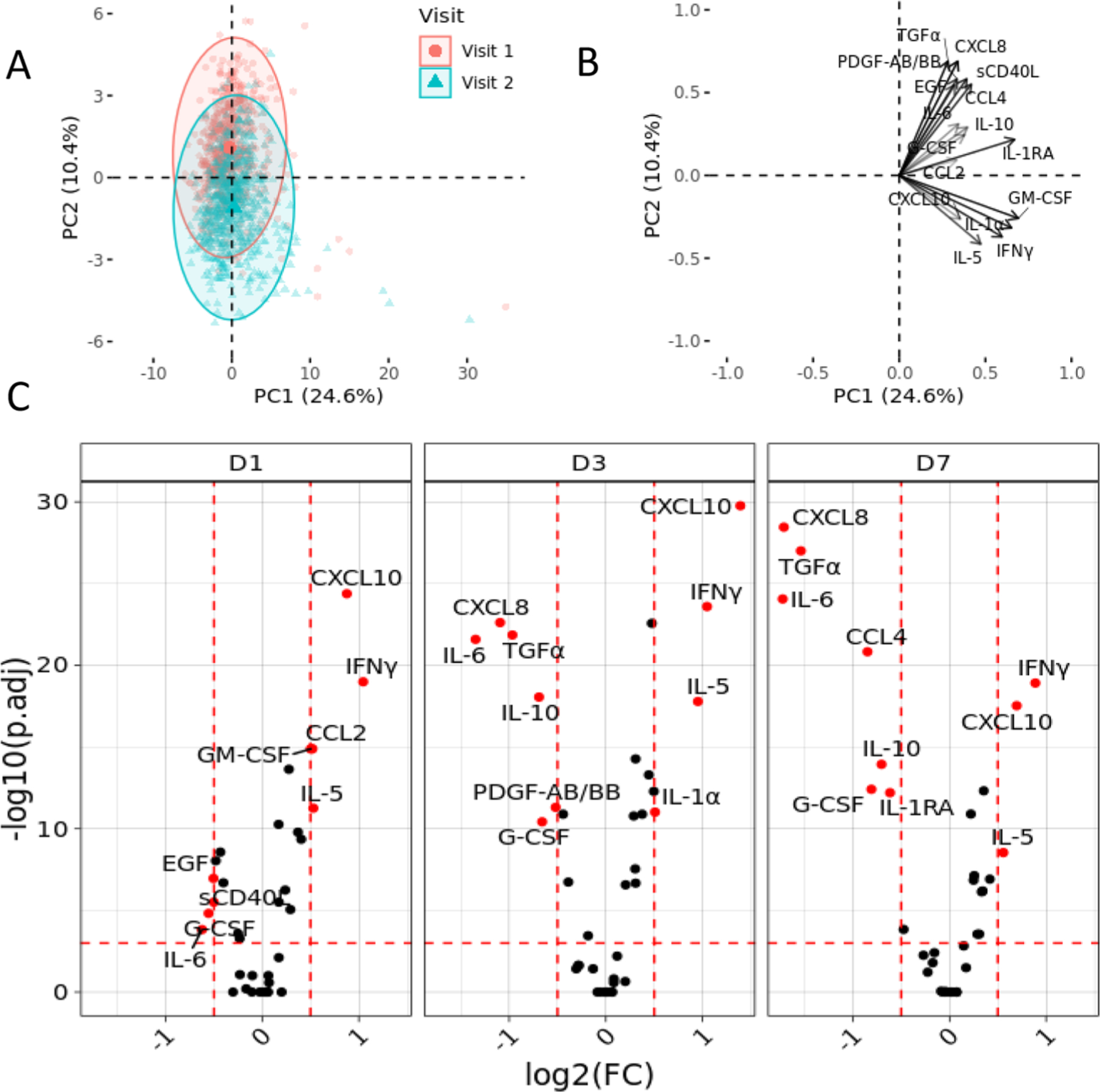
Ontogeny of plasma cytokines and chemokines over the first week of life. A. Principal component analysis (PCA) demonstrating ontogeny of plasma cytokine and chemokines. PCA was used to plot log_10_-transformed plasma cytokine/chemokine concentrations and revealed sample clustering by Visit (age) in EPIC-HIPC Gambia cohort. B. Cytokine loadings on principal components 1 and 2. C. Volcano plots provide further resolution of the ontogeny of cytokine and chemokines. Volcano plots with cytokine/chemokines significantly increased over day of life (DOL) 0 in EPIC-HIPC Gambia cohort across the first week of life. Significant cytokines or chemokines (p-value < 0.01, absolute log2 [fold change vs DOL0] > 0.2) are marked in red with abbreviated cytokine/chemokine names. A paired Wilcoxon was used to test for cytokine significance. (n = 608 participants for Visit 1 and Visit 2: 202 DOL1, 206 DOL3, 200 DOL7).

To identify potential ontogenetic changes, we measured the concentration of the plasma cytokines and chemokines in 608 healthy Gambia mother-newborn pairs (n = 202 at DOL1, n = 206 at DOL3, n = 200 at DOL7) over the first week of life. Principal component analysis (PCA) demonstrated the expression of cytokines and chemokines. The first two principal components (PC1 and PC2) accounted for 35.4% of total variance (Fig. 1A). PC2 captures the differences between Visit 1 and Visit 2, accounts for ~10% of total variance. PC3 also contained some variance information from ontogenetic changes between Visit 1 and Visit 2 (Supp Fig. 1A; ~6.6% of total variance). The PCA loadings illustrate that TGFα, CXCL8, sCD40L, CCL4, IL-6, and PDGF-AB/BB are elevated at birth (Visit 1) relative to Visit 2 (DOL 1, 3, or 7). Fig. 1B lists the top 15 contributing cytokines and chemokines, while Supp Fig. 1B demonstrates all cytokines and chemokines.

### 3.4. Increase of plasma cytokine and chemokine ontogeny changes with the increase of age

We tested whether the relative abundances of individual cytokines and chemokines changed across the first week of life (Fig. 1C; Supplementary Table 1). At DOL1, plasma concentrations of CXCL10, IFNγ, CCl2, and IL-5 were all significantly higher over DOL0, while EGF, sCD40L, G-CSF, and IL-6 are significantly lower. DOL3 revealed more pronounced differences from DOL0, with greater increases in CXCL10, IFNγ, IL-5, and IL-1α, and a downregulation of CXCL8, TGFα, IL-6, IL-10, PDGF-AB/AA, and G-CSF. Lastly, at DOL7, IFNγ, CXCL10, and IL-5 continue to be significantly upregulated, while CXCL8, TGFα, IL-6, CCL4, IL-10, IL-1RA, and G-CSF, were significantly downregulated.

### 3.5. Plasma cytokine and chemokine cluster by time series analysis during the first week of life

**Fig. 2.**
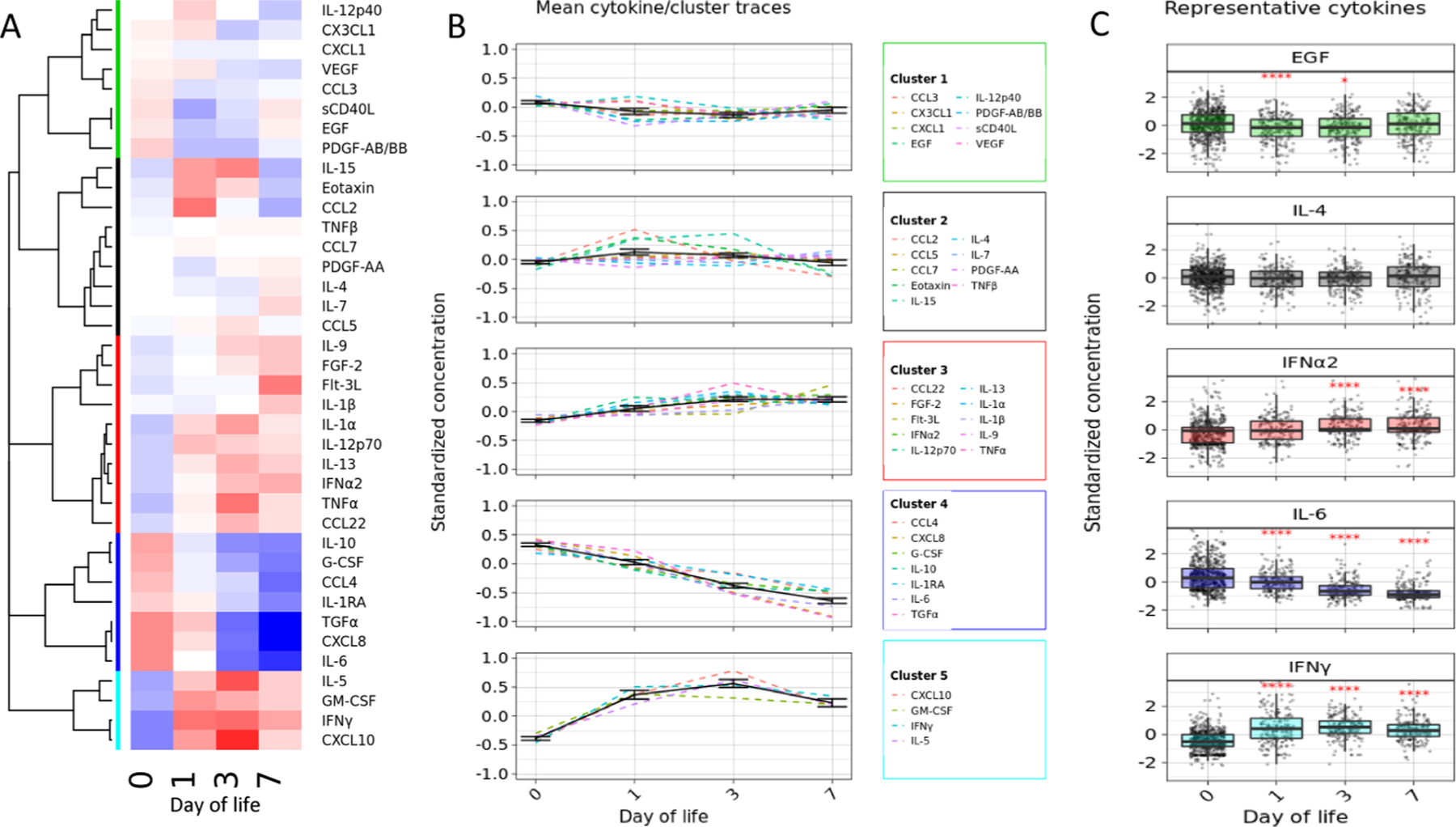
Time series analysis across the first week of life demonstrates distinct cytokine/chemokine cluster patterns. A. Time series clustering. Hierarchical clustering was performed to group cytokines and chemokines based on their mean time series. B. Cytokine traces by cluster. The mean cluster (solid line) and cytokine (dashed line) traces for each day of life (DOL) show consistent trajectories within each cluster across the first week of life. Error bars represent 95% confidence intervals for the cluster mean. The cytokines in clusters 1 and 2 exhibit small changes over the first week of life, with some reaching transient troughs or peaks at DOLs 1 and 3. The cytokines in cluster 3 rise slowly across the first week of life. Cluster 4, which primarily contains inflammation-associated cytokines such as IL-6 and IL-10, shows a steady decrease across the first week of life. The cytokines in cluster 5, including IFNγ and CXCL10, increase rapidly between birth and DOL1, then peak and reverse course by DOL3. C. Representative cytokines were chosen for each cluster to show the variation within groups. Statistical comparison employed T-tests on standardized cytokine concentrations for paired samples between DOL0 and the follow-up visit. (****: p < 0.0001, ***: p < 0.001, **: p < 0.01, *: p < 0.05. n = 608: 202 DOL1, 206 DOL3, 200 DOL7.)

Time series clustering highlighted specific patterns of plasma cytokine and chemokine concentrations over the first week of life (Fig. 2). Hierarchical clustering of mean standardized, dynamic time warped cytokine and chemokine profiles across the first week of life revealed five clusters of cytokines (Fig. 2B, Supp Fig. 2). The trajectories of these clusters are shown in Fig. 2B, along with the mean trajectories of their component cytokines and chemokines. Error bars represent 95% confidence intervals for the cluster mean (Supp Table 1). Paired t-tests were performed to test whether the cluster concentrations were significantly different between DOL0 and subsequent days of life (Supp Fig. 2).

As a sensitivity analysis, we compared our cluster method to an independent validation method[43]. We compared the consensus values of both methods, which can be interpreted as the frequency that cytokines are placed into the same cluster across repeated runs[43]. The average consensus for clusters 1–5 was 79.4% using the main methodology and 53.9% using the validation methodology.

Representative cytokines and chemokines for each cluster are shown in Fig. 2C to illustrate the variation within groups. Paired t-tests of cytokine concentrations between DOL0 and the follow-up visits were employed to assess for significance. The detailed pattern of each cytokine and chemokine was analysed as standardized log_10_-as well as raw-concentrations and depicted in Supplementary Fig. 2A and 2B, respectively. This analysis suggested that there are distinct trajectories for different groups of cytokines and chemokines during the first week of life.

## 4. Discussion

In this study, plasma cytokines and chemokines were measured in a longitudinal cohort of healthy, full-term Gambian newborns throughout the first week of life, using highly standardized, quantitative multiplex protein assays. A variety of longitudinal changes were observed. Plasma concentrations of CXCL10, GM-CSF, IFNγ, and IL-5 increased over the first week of life while those of CCL4, CXCL8, G-CSF, IL-10, IL-1RA, IL-6, and TGFα decreased. We thus observed that ontogeny is a strong driver of human neonatal plasma cytokine and chemokine concentrations.

Cytokines and chemokines are key signaling proteins that act via cognate receptors to shape immune responses [6]. In early life, cytokines and chemokines play a crucial role in the maturation and development of lymphoid progenitors [44]. Our findings indicate a significant increase of Th1 cytokines and a significant decrease of pro-inflammatory and regulatory cytokines and chemokines across the first week of life. We observed a significant increase of Th1-polarizing cytokines, including IFNγ, IFNγ-inducible CXCL10 (IP10), GM-CSF, which may contribute to progressive development of cell mediated immunity with age, as well as IL-5, a Th2-polarizing cytokine that contributes to eosinophil activation. Plasma IFNγ concentrations rose significantly though modestly across the first week of life, along with those of IL-12 and TNFα, consistent with the generally tight regulation of cytokines and limited ability of human neonatal leukocytes to produce these cytokines [33,45,46]. Of note, IFNγ is important to early life host defense [47] as: 1) low birthweight newborns who are highly susceptible to infection are relatively deficient in IFNγ production[48]; 2) Addition of IFN-γ to human newborn blood in vitro enhanced innate immune function including leukocyte responses to bacteria and plasma opsonophagocytic activity; and 3) IFNγR deficient children present in early life with severe mycobacterial infections[49]. Thus the rise of IFN and IFN-associated CXCL10 can be viewed as a gradual ontogenetic enhancement in the ability to confront intracellular pathogens.

Newborns emerge out of a highly regulated maternofetal environment aimed at protecting non-self (the fetus) from self, the maternal immune system, while in utero. For example, the placenta produces mediators such as progesterone and prostaglandin to promote a Th2 bias in the fetus [50,51]. Human neonatal antigen-presenting cells demonstrate reduced stimulus-induced production of Th1- and Th17 -type cytokines coupled with preserved production of Th2 -type cytokines, contributing to an anti-inflammatory environment in utero that reduces the risk of abortion or pre-term delivery[46].

Although the in utero environment is highly tolerogenic, due to a variety of immunosuppressive mechanisms [52–54], birth itself is a controlled inflammatory process. Collaboration between innate and adaptive immune responses is required to sustain pregnancy [55], and disturbance of this balance occurs during physiological labor. In contrast to the Th1-type cytokine increase, we found a significant decrease of CCL4, CXCL8, IL-6, IL-1RA, G-CSF, IL-10, and TGFα over the first week of life. A decline in pro-inflammatory and pro-resolution cytokines and chemokines including CXCL8, CCL4, and IL-6 [56], along with antiinflammatory factors including IL-10 and TGFα, might reflect resolution of the birthing process.

After birth, the immune system begins to transition towards a complex and microbe-rich extra-uterine environment. The establishment of the gut microbiome begins at birth[57] and continues to develop until ~3 years of age [58–60, and maintains interplay with the developing immune system[61]. The increase in plasma concentrations of IFNα2, IL-12p70, IL-13, IL-1a, IL-9, and TNFα support an ontogenybased transition away from the highly regulated maternofetal environment towards the more diverse ex utero environment. A previous study looking at 24 matched samples, found no difference between maternal blood and newborn cord blood from elective Cesarean deliveries with respect to plasma concentrations of IL-6, CXCL-8, IL-10, and IL1-RA (PMID 26136749). Our study, focused on vaginal deliveries, was not designed to correlate maternal cytokine levels with that of the neonates, though based on the aforementioned study we hypothesize that cytokine concentrations at birth likely correlate with those in maternal plasma. Of note, plasma concentrations of certain cytokines such as IL-6 are higher in vaginal vs. C-section delivery[29].

We did not observe any significant differences in cytokine concentrations between infants who were breastfed vs those who were formula feed (data not shown). Despite this observation, it remains possible that breast milk cytokine concentrations may impact those of in infant plasma[62,63], as: 1) analysis of plasma cytokines and chemokine concentrations beyond the first week of life may demonstrate differences between breastfed and non-breastfed levels; 2) detecting potential differences in plasma cytokines due to breastfeeding may require specific focus on mucosal immunity/oral tolerance, as has been reviewed[64], and correlations with maternal plasma cytokine/chemokine concentrations as well as maternal breastmilk cytokine/chemokine concentrations that differ by parity status[62]. Overall, there is need for additional studies on the relation of method of delivery[29], breast-vs formula-feeding, and early life immune ontogeny.

Consistent with other studies of cytokines and chemokines in early life [34,65], we did not observe sex-related differences in plasma cytokine and chemokine concentrations. Previous studies in healthy children >1 month old have found age- and sex-differences in plasma cytokines and chemokines, potentially due to sex hormones [16,18,23,66]. Given that there are age-dependent sex differences later in life [67,68], future studies should investigate at which timepoint these sex-dependent cytokine changes occur and whether any such changes correspond to sex-specific differences in innate and adaptive immunity.

Given growing evidence of rapid changes in infant immunity [40], our study was designed to capture clinical and cytokine/chemokine data from the day of birth. Cytokines and chemokines in Cluster 1 (CCL3, CX3CL1, CXCL1, EGF, IL-12p40, PDGF-AB/BB, sCD40L, VEGF) and Cluster 2 (CCL2, CCL5, CCL7, Eotaxin, IL-15, IL-4, IL-7, PDGF-AA, TNFβ) play a crucial role in activating or mediating the host immune response to pathogens. These changes over the first week of life illustrate the dynamic nature of the early life immune system. Several reviews detail the function of each of these cytokines and chemokines [16,18,19–23].

Our results have potential translational applications. Compared to adults, infants have increased susceptibility to infection, yet most preventative strategies for neonates rely upon our understanding of the adult immune system. Inflammatory protein markers such as those regulated by IL-6, like C-reactive protein (CRP), are important clinical analytes for early onset neonatal sepsis [69,70]. In infants, G-CSF, IL-6, CXCL8 (IL-8), and TNF-α have been identified as potential markers of Gram-negative bacteremia in the neonatal intensive care unit [71]. There remains an unmet need to define effective biomarkers of early onset sepsis, defined as less than 72 h in pre-term infants, and less than 7 days in term infants [69,72].

The age-dependent changes observed here suggest that plasma cytokines and chemokines have day of life-specific normal ranges, with future studies needed to further validate this finding. We observed a clear plasma cytokine/chemokine ontogeny signature during the first week of life. As cytokines, such as IFN-γ and IL-1β, can contribute to “innate memory” or trained immunity [73 74 75], future studies should investigate the relationship of these plasma cytokines, both at baseline and after infection or immunization, on measures of trained immunity. Indeed, characterizing immune trajectories in healthy newborns will help address questions concerning how distinct early life immunity shapes susceptibility to disease.

Our study also has a number of limitations. Although the number of infants included in this study is substantial, samples were collected at a single study site in The Gambia (West Africa), requiring further studies to assess the generalizability of these findings. Nevertheless, our observation of significant changes in the cytokine IFNγ and related chemokine, the IFNγ-inducible CXCL10, with similar trajectories noted in geographically and genetically distinct newborns in Papua New Guinea [39] suggests that the patterns we observed have broader applicability. Secondly, we do not yet know whether the observed trajectories remain consistent between the first week of life and the remainder of the vulnerable neonatal period, defined as first 28 days of life. This is a key question in the field of immune ontogeny and will require further study.

## 5. Conclusion

Our findings demonstrate an age-dependent pattern of plasma cytokines and chemokines in healthy, term Gambian infants, representing an ontogenetic trajectory reflective of the initiation of a post-birth transition from a Th2-polarized to a more balanced Th1/Th2 immune state. Our findings help define the baseline trajectories of plasma cytokines and chemokines in healthy newborns and will inform future studies to correlate plasma cytokines and chemokines with responses to immune challenge such as immunization, as well as deviations from healthy trajectories due to infection or other diseases.

## Supplementary Material

Supp.materials

## Figures and Tables

**Table 1:** Demographic characteristics of the study participants.

Characteristics	Frequency (n)	Percent (%)
**Sex of Baby:**		
Female/Male	300/308	49.3/50.7
**Birth weight (kg):**		
Avg (±SEM)	3.16 ± 0.015	
**Age of mothers (years):**		
18–19 years	26	4.3
20–24 years	135	22.2
25–29 years	177	29.1
30–34 years	126	20.7
35–39 years	109	17.9
40–45 years	35	5.8
**Term of Baby:**		
Preterm (<37 weeks)	4	0.7
Early term (37–38 weeks)	150	24.7
Full term (39–40 weeks)	374	61.5
Late term (>40 weeks)	80	13.2
**Ethnicity of Baby:**		
Mandinka	291	47.9
Jola	96	15.8
Fula	79	13.0
Wollof	68	11.2
Serahule	22	3.6
Others	52	8.6
**Frequency of breastfeeding:**		
Visit 1 (Yes/No)	538/70	88.5/11.5
Visit 2 (Yes/No)	601/7	98.8/1.2

## Abbreviations:

DOL: day of life
EPIC: Expanded Program on Immunization Consortium
HIPC: Human Immune Project Consortium
Ig: immunoglobulin
IL: interleukin
TGF-β: transforming growth factor beta
TNF: tumor necrosis factor
CXCL: C-X-C motif ligand
CCL: CC chemokine ligands
VEGF: vascular endothelial growth factor
IFN: interferon
G-CSF: granulocyte colony-stimulating factor
GM-CSF: granulocyte-macrophage colony-stimulating factor
FGF: fibroblast growth factor
Treg: Regulatory T cell
