# Supplementary material for: Ontogeny of plasma cytokine and chemokine concentrations across the first week of human life
Source: Cytokine. Author manuscript; Available in PMC 2021 Dec 11. (PMC8665647; doi:10.1016/j.cyto.2021.155704)
Supplement: Supp.materials [file NIHMS1744266-supplement-Supp_materials.pptx]

## Slide 1
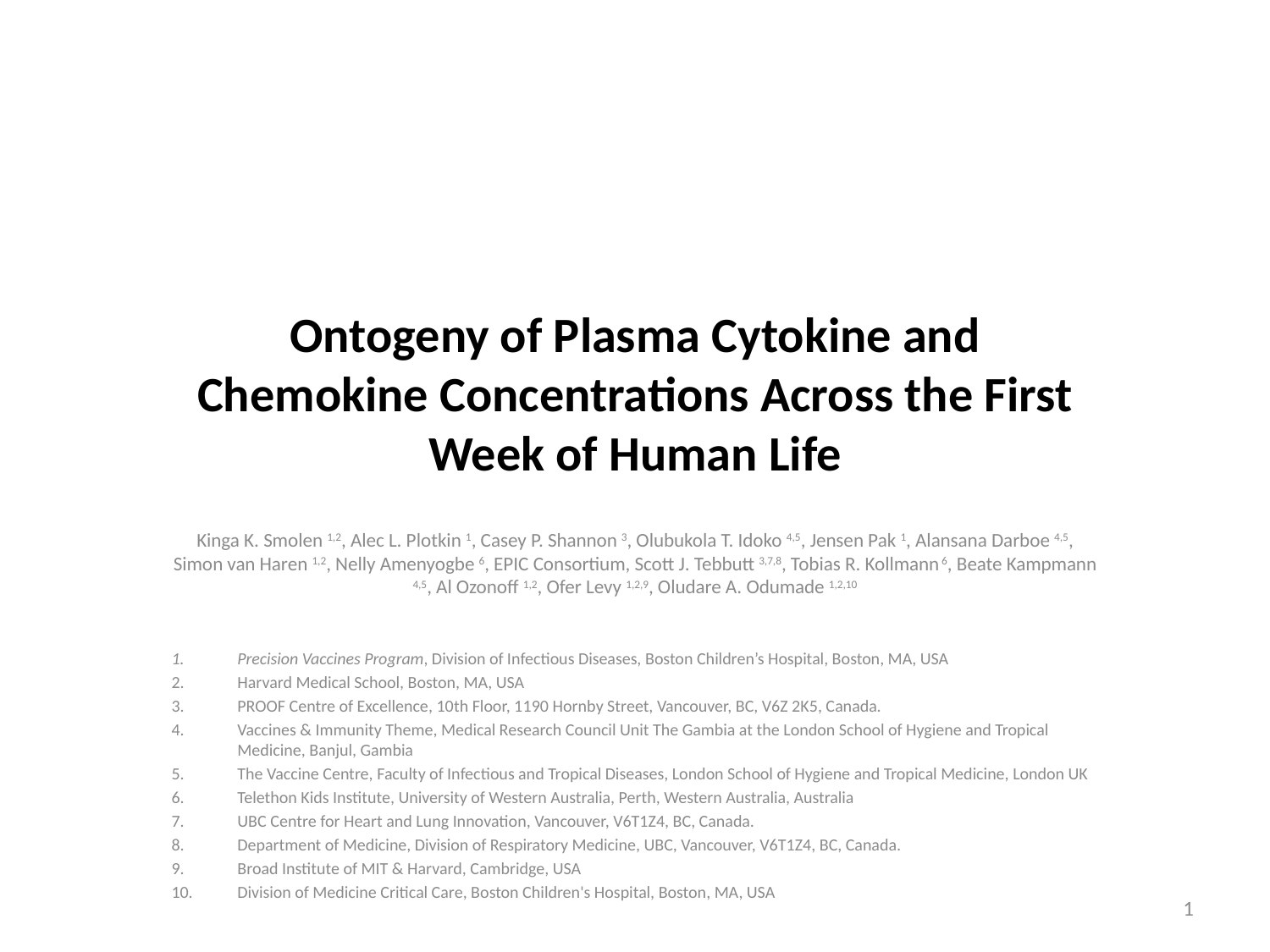

# Ontogeny of Plasma Cytokine and Chemokine Concentrations Across the First Week of Human Life
Kinga K. Smolen 1,2, Alec L. Plotkin 1, Casey P. Shannon 3, Olubukola T. Idoko 4,5, Jensen Pak 1, Alansana Darboe 4,5, Simon van Haren 1,2, Nelly Amenyogbe 6, EPIC Consortium, Scott J. Tebbutt 3,7,8, Tobias R. Kollmann 6, Beate Kampmann 4,5, Al Ozonoff 1,2, Ofer Levy 1,2,9, Oludare A. Odumade 1,2,10
Precision Vaccines Program, Division of Infectious Diseases, Boston Children’s Hospital, Boston, MA, USA
Harvard Medical School, Boston, MA, USA
PROOF Centre of Excellence, 10th Floor, 1190 Hornby Street, Vancouver, BC, V6Z 2K5, Canada.
Vaccines & Immunity Theme, Medical Research Council Unit The Gambia at the London School of Hygiene and Tropical Medicine, Banjul, Gambia
The Vaccine Centre, Faculty of Infectious and Tropical Diseases, London School of Hygiene and Tropical Medicine, London UK
Telethon Kids Institute, University of Western Australia, Perth, Western Australia, Australia
UBC Centre for Heart and Lung Innovation, Vancouver, V6T1Z4, BC, Canada.
Department of Medicine, Division of Respiratory Medicine, UBC, Vancouver, V6T1Z4, BC, Canada.
Broad Institute of MIT & Harvard, Cambridge, USA
Division of Medicine Critical Care, Boston Children's Hospital, Boston, MA, USA
1

## Slide 2
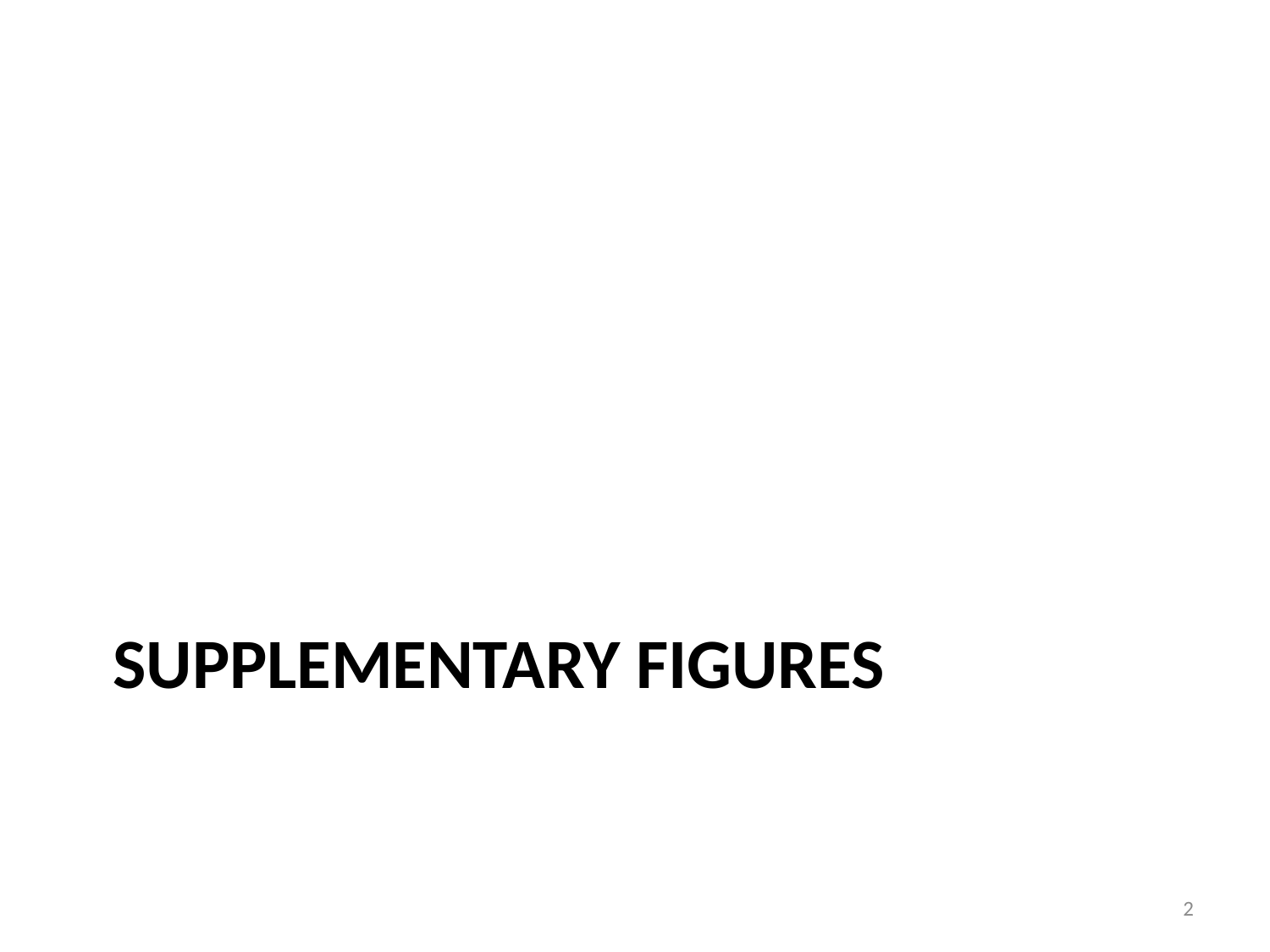

# Supplementary Figures
2

## Slide 3
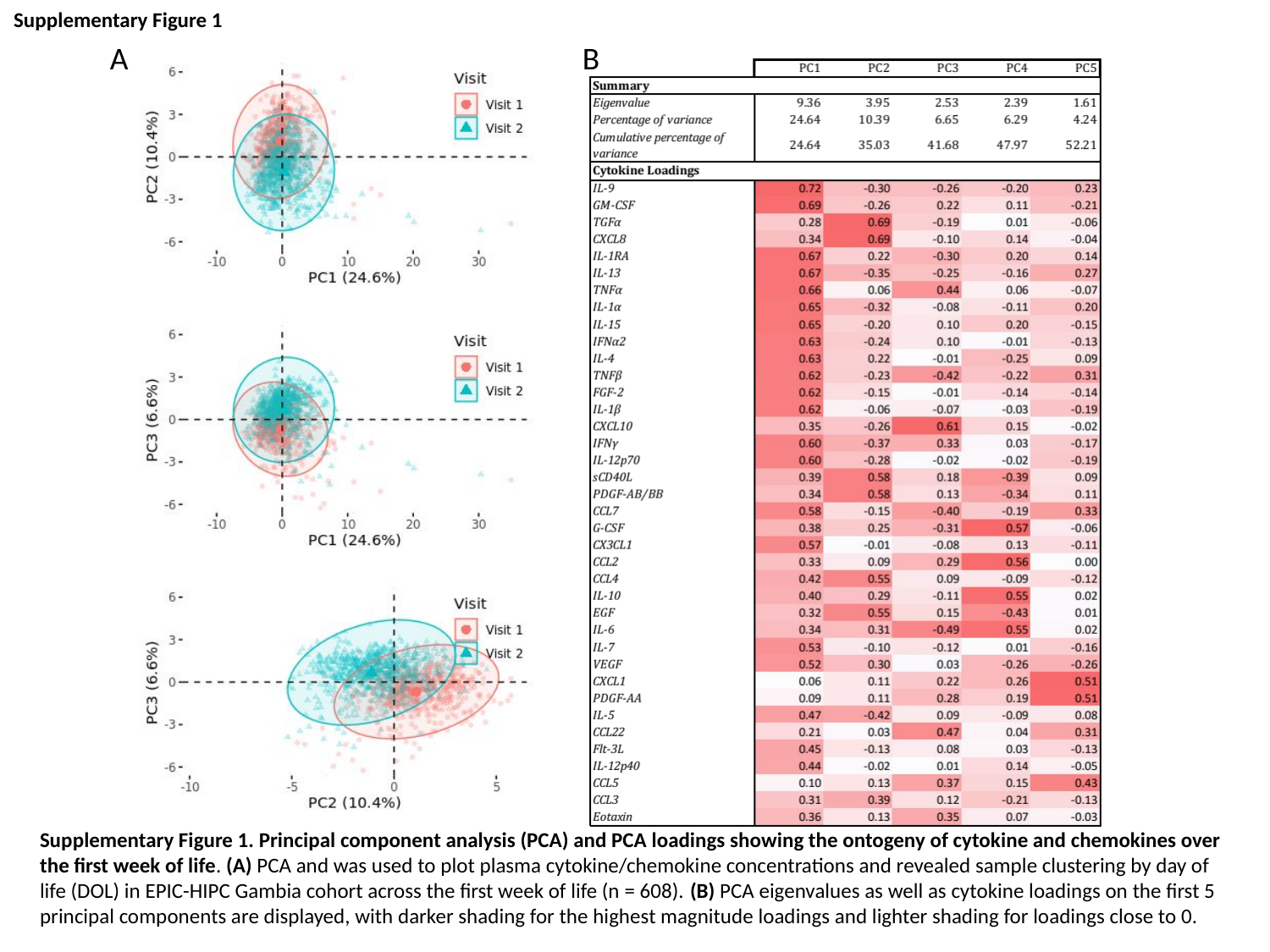

Supplementary Figure 1
A
B
Supplementary Figure 1. Principal component analysis (PCA) and PCA loadings showing the ontogeny of cytokine and chemokines over the first week of life. (A) PCA and was used to plot plasma cytokine/chemokine concentrations and revealed sample clustering by day of life (DOL) in EPIC-HIPC Gambia cohort across the first week of life (n = 608). (B) PCA eigenvalues as well as cytokine loadings on the first 5 principal components are displayed, with darker shading for the highest magnitude loadings and lighter shading for loadings close to 0.

## Slide 4
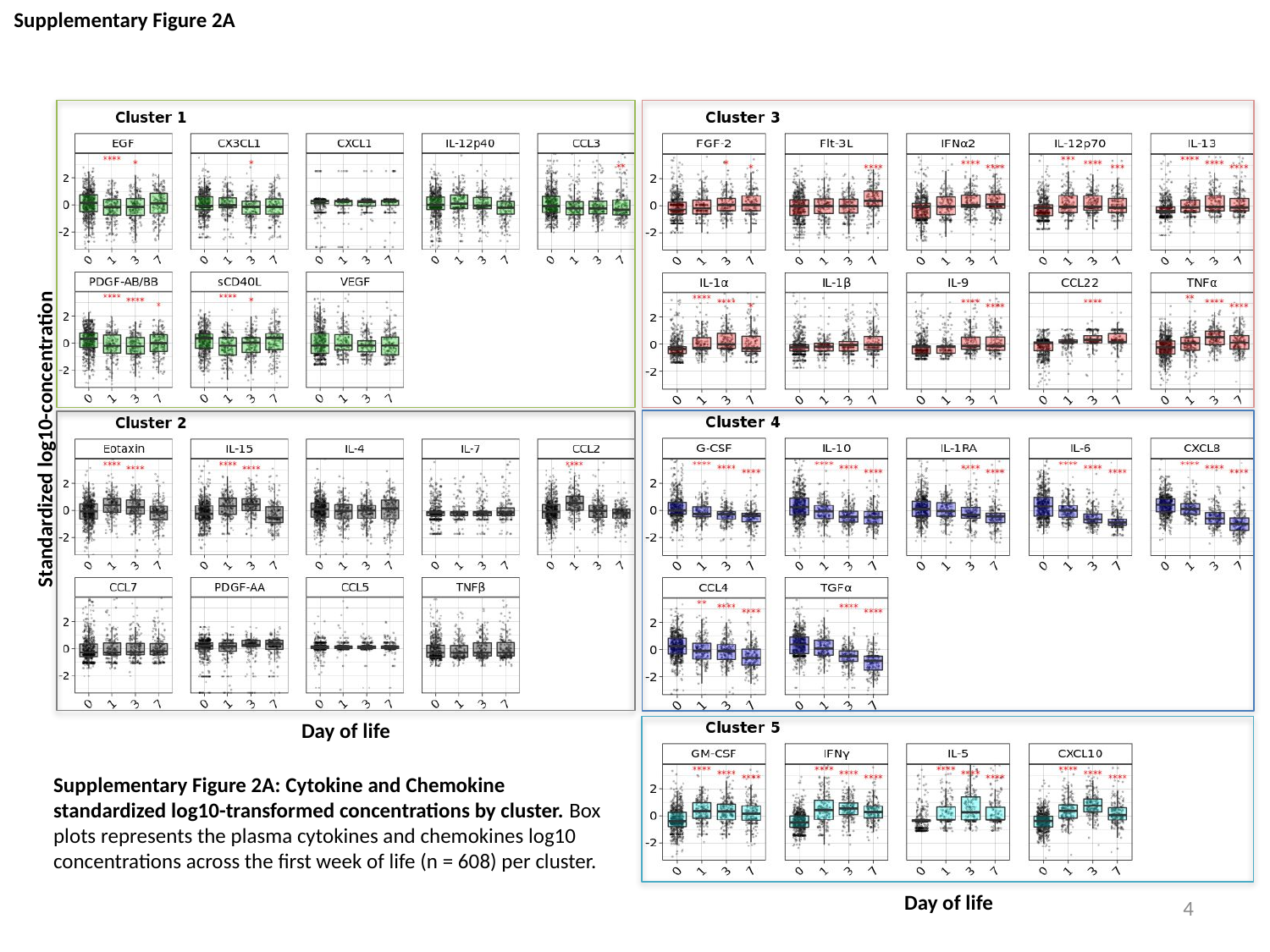

Supplementary Figure 2A
Standardized log10-concentration
Day of life
Day of life
Supplementary Figure 2A: Cytokine and Chemokine standardized log10-transformed concentrations by cluster. Box plots represents the plasma cytokines and chemokines log10 concentrations across the first week of life (n = 608) per cluster.
4

## Slide 5
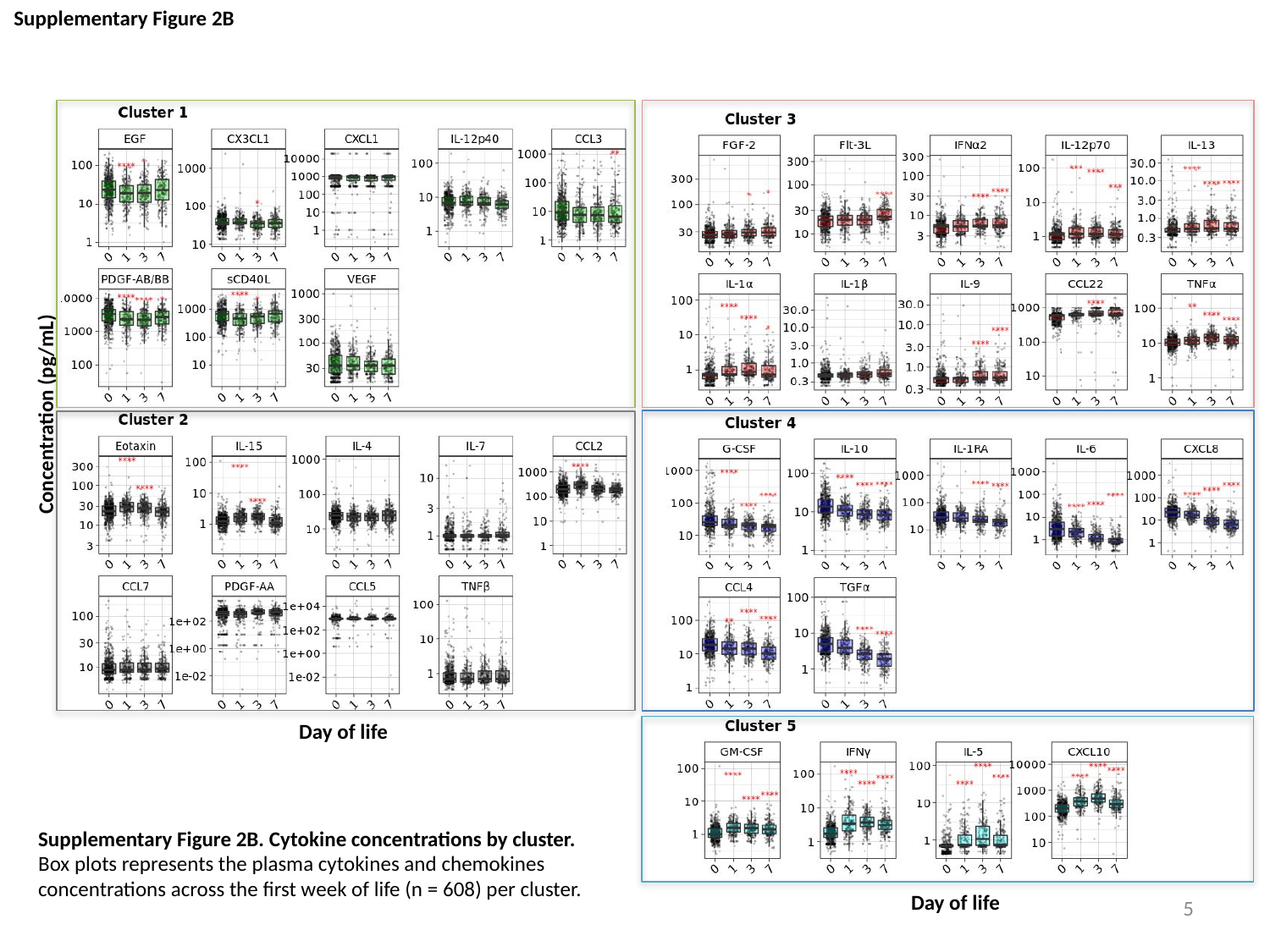

Supplementary Figure 2B
Concentration (pg/mL)
Day of life
Day of life
Supplementary Figure 2B. Cytokine concentrations by cluster. Box plots represents the plasma cytokines and chemokines concentrations across the first week of life (n = 608) per cluster.
5

## Slide 6
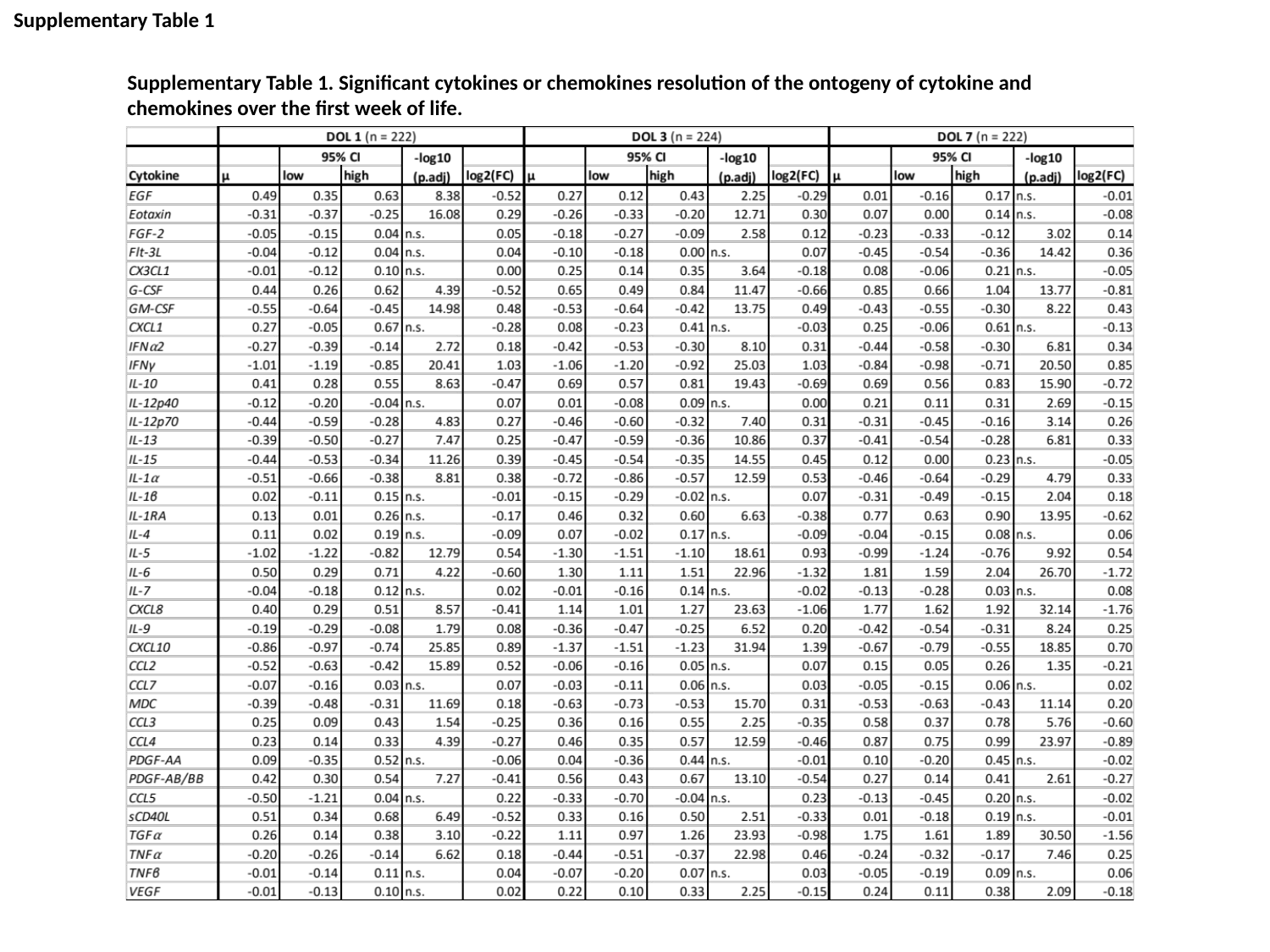

Supplementary Table 1
Supplementary Table 1. Significant cytokines or chemokines resolution of the ontogeny of cytokine and chemokines over the first week of life.
